# Data set describing the *in vitro* biological activity of JMV2009, a novel silylated neurotensin(8–13) analog

**DOI:** 10.1016/j.dib.2020.105884

**Published:** 2020-06-20

**Authors:** Élie Besserer-Offroy, Pascal Tétreault, Rebecca L Brouillette, Adeline René, Alexandre Murza, Roberto Fanelli, Karyn Kirby, Alexandre Parent, Isabelle Dubuc, Nicolas Beaudet, Jérôme Côté, Jean-Michel Longpré, Jean Martinez, Florine Cavelier, Philippe Sarret

**Affiliations:** a Department of Pharmacology-Physiology, Faculty of Medicine and Health Sciences, Université de Sherbrooke, Sherbrooke, Québec, Canada; b Department of Anaesthesiology, Faculty of Medicine and Health Sciences, Université de Sherbrooke, Sherbrooke, Québec, Canada; c Institut de pharmacologie de Sherbrooke, Université de Sherbrooke, Sherbrooke, Québec, Canada; d Centre de recherche du Centre hospitalier universitaire de Sherbrooke, CIUSSS de l'Estrie - CHUS, Sherbrooke, Québec, Canada; e Institut des Biomolécules Max Mousseron, UMR-5247, CNRS, Université Montpellier, ENSCM, Montpellier, France; f Department of Pharmacy, Faculty of Medicine and Pharmacy, Université de Rouen, Mont-Saint-Aignan, France

**Keywords:** Unnatural amino-acid, G protein-coupled receptor, Neurotensin, G protein, Arrestin

## Abstract

Neurotensin (NT) is a tridecapeptide displaying interesting antinociceptive properties through its action on its receptors, NTS1 and NTS2. Neurotensin-like compounds have been shown to exert better antinociceptive properties than morphine at equimolar doses. In this article, we characterized the molecular effects of a novel neurotensin (8–13) (NT(8–13)) analog containing an unnatural amino acid. This compound, named JMV2009, displays a Silaproline in position 10 in replacement of a proline in the native NT(8–13). We first examined the binding affinities of this novel NT(8–13) derivative at both NTS1 and NTS2 receptor sites by performing competitive displacement of iodinated NT on purified cell membranes. Then, we evaluated the ability of JMV2009 to activate NTS1-related G proteins as well as to promote the recruitment of β-arrestins 1 and 2 by using BRET-based cellular assays in live cells. We next assessed its ability to induce p42/p44 MAPK phosphorylation and NT receptors internalization using western blot and cell-surface ELISA, respectively. Finally, we determined the *in vitro* plasma stability of this NT derivative. This article is associated with the original article “Pain relief devoid of opioid side effects following central action of a silylated neurotensin analog” published in *European Journal of Pharmacology*[1]. The reader is directed to the associated article for results interpretation, comments, and discussion.

**Specifications Table**

**Table:** 

**Subject**	Pharmacology
**Specific subject area**	*In vitro* and *in cellulo* characterization of JMV2009, a neurotensin(8–13) analog, on NTS1 and NTS2
**Type of data**	Chemical structure Figure Graph Table
**How data were acquired**	Radioligand binding BRET-based assays for activation of G proteins and β-arrestins recruitment Cell-surface ELISA for internalization of NT receptors Western blot for the activation of p42/p44 *Exvivo* plasma stability of JMV2009 **Instruments:** PerkinElmer Wizard^2^ 1470 γ-counter Tecan Genios Pro multimode plate reader Waters UPLC system coupled with a SQ detector 2 and a PDA eλ detector Waters Acquity CSH C18 column, 2.1 mm X 50 mm, 1.7 µm spherical size Micromass Platform II quadrupole mass spectrometer (Micromass) fitted with an electrospray source coupled with a Waters HPLC Waters Delta-Prep 4000 equiped with a Waters 486 UV detector Delta-Pak C18 column (40 × 100 mm, 15 µm, 100 Å)
**Data format**	Raw Analysed
**Parameters for data collection**	All parameters of data collection are reported in Section 2. Experimental Design, Materials, and Methods.
**Description of data collection**	Radioactivity counts retained on GF/C filters were counted on a γ-counter. Filtered luminescence readings of BRET experiments were recorded in endpoint readout using a multimode plate reader equipped with a BRET2 filter set. Optical density (absorbance) of the colorimetric reaction for cell-surface ELISA was recorded in endpoint readout using a multimode plate reader using a 450 nm filter. Western blots for phosphorylation of p42/p44 were revealed using an enhanced chemiluminescence detection with high sensitivity films.
	Remaining intact peptide in plasma stability assay was quantified using an internal standard and UPLC/MS system. Graphs, data normalization, and non-linear regression fits were done using GraphPad Prism v7.0a. Western blots for phosphorylation of p42/p44 were revealed using an enhanced chemiluminescence detection with high sensitivity films. Remaining intact peptide in plasma stability assay was quantified using an internal standard and UPLC/MS system. Graphs, data normalization, and non-linear regression fits were done using GraphPad Prism v7.0a.
**Data source location**	Institut de pharmacologie de Sherbrooke, Université de Sherbrooke Sherbrooke, Québec, Canada J1H5N4
**Data accessibility**	Repository name: Figshare Data identification number: 10.6084/m9.figshare.11962689 Direct URL to data: https://doi.org/10.6084/m9.figshare.11962689
**Related research article**	Tétreault P, Besserer-Offroy É, Brouillette RL, René A, Murza A, Fanelli R, Kirby K, Parent A, Dubuc I, Beaudet N, Côté J, Longpré JM, Martinez J, Cavelier F, Sarret P. Pain relief devoid of opioid side effects following central action of a silylated neurotensin analog. *Eur. J. Pharmacol.*, **882**, 2020, 173174.

## Value of the Data

• These data characterize the *in vitro* and *in cellulo* behavior of a novel neurotensinergic compound with analgesic properties.
• These data provide insights into different G protein activation and β-arrestin recruitment on the NTS1 receptor and functional assay on the NTS2 receptor including p42/p44 phosphorylation and receptor internalization.
• These data provide insights into the molecular mechanisms underlying the action of JMV2009

## 1 Data description

This article describes the data that are analysed, interpreted, and discussed in Tétreault et al. [1]. Raw data are made freely available at https://doi.org/10.6084/m9.figshare.11962689.

### 1.1 JMV2009 synthesis and chemical characterization

**Scheme 1:**
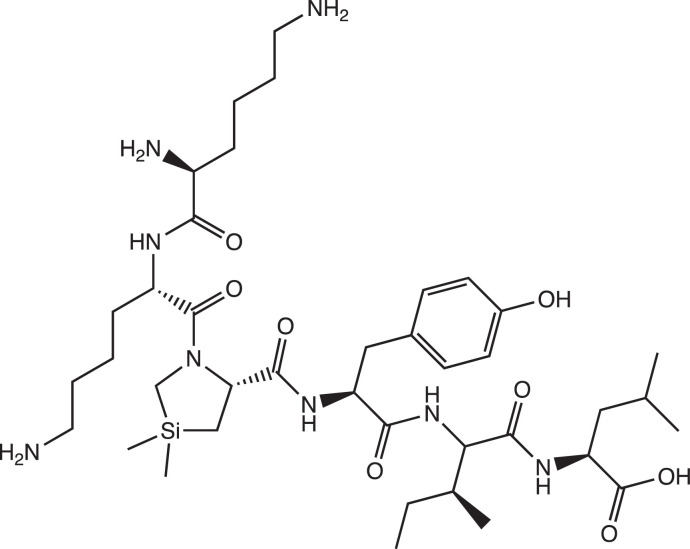
Chemical structure of JMV2009.

**Fig. 1:**
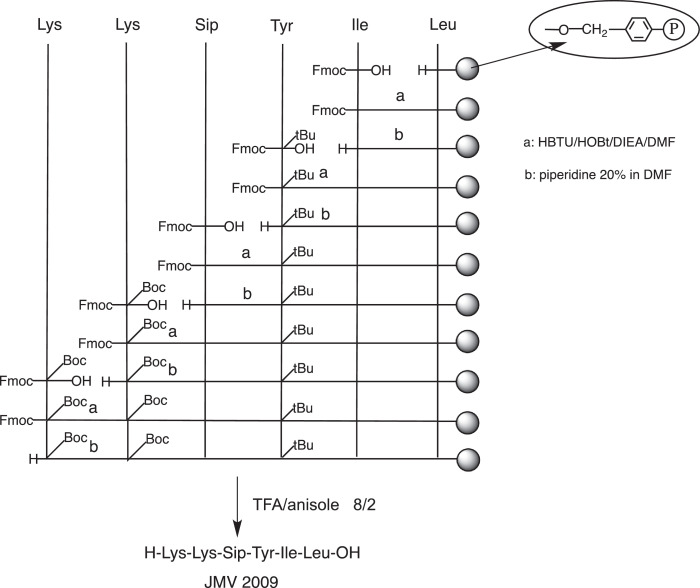
Synthetic procedure for the hexapeptide JMV2009.

**Fig. 2:**
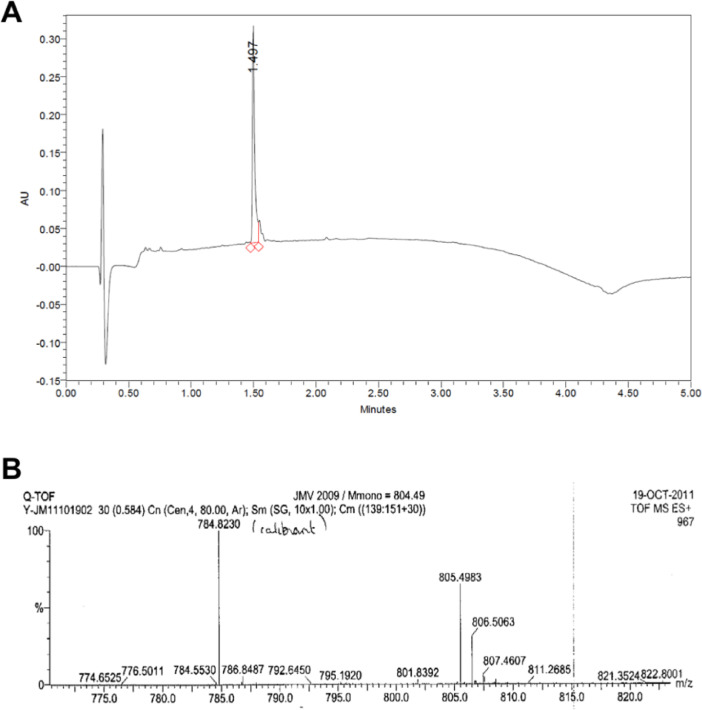
Chemical characterization of JMV2009. (A) HPLC-UV spectra of JMV2009 for purity characterization. (B) HRMS spectra of JMV2009 for exact mass determination, peak at 784.8230 Da is an internal calibrator for the HRMS.

The hexapeptide JMV2009 (Scheme 1) was synthesized by solide-phase method using Wang resin preloaded with Leucine residue (Fig. 1) as described in Section 2.2. below. The 9-fluorenylmethyloxycarbonyl (Fmoc) protection was used as temporary protection of the N-terminal amino groups, and N‑*tert-*Butyloxycarbonyl (Boc) and *tert*-Butyl (tBu) were used as orthogonal side-chain protections. Couplings of protected amino acids were carried out with a solution of HBTU/HOBt reagents. The unnatural amino acid Silaproline (Sip) has been synthetized as previously described [2,3], Fmoc-protected, and incorporated in the automated synthesis as other natural amino acid. The use of Wang resin allowed peptide release from the resin and the deprotection of side chains of the desired protected peptide with TFA in the presence of anisole as scavenger. The resulting peptide JMV 2009 was purified by preparative reverse-phase HPLC on a C_18_ column and its purity and structure were confirmed by HPLC-UV and ESI mass spectrometry, respectively (Fig. 2).

### 1.2 JMV2009 binding at NT receptors

**Fig. 3:**
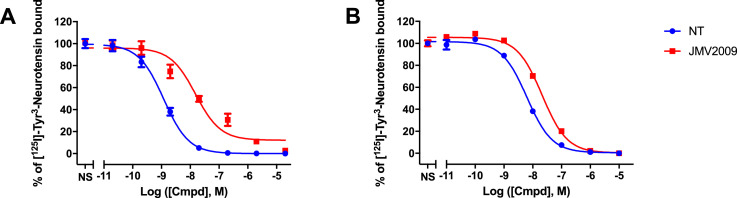
Concentration-displacement curves of NT and JMV2009. Displacement of [^125^I]-Tyr^3^-Neurotensin by NT and JMV2009 on cell membranes expressing hNTS1 (A) or hNTS2 (B).

We first evaluated the binding affinities of this new analog on both NTS1 and NTS2 receptors. Binding experiments of neurotensin (NT) and JMV2009 were carried out on freshly prepared membranes of CHO-K1 cells expressing the human NTS1 receptor or 1321N1 cells expressing the human NTS2 receptor as previously described [4]. Concentration-displacement curves (Fig. 3) were used to fit a non-linear regression model in Graphpad Prism and determine the IC_50_ values for NT and JMV2009 (Table 1).

**Table 1:** Binding affinities of NT and JMV2009.

	IC_50_ NTS1, nM	IC_50_ NTS2, nM
**NT**	1.2 ± 0.2	6.2 ± 0.5
**JMV2009**	15.2 ± 4.7	21.2 ± 1.9

Values are expressed as IC_50_ ± SEM of at least three independent determinations.

### 1.3 JMV2009 plasma stability

**Fig. 4:**
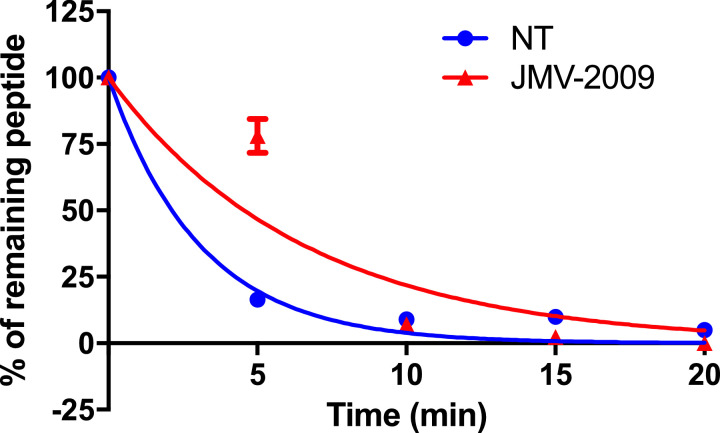
Plasma stability of JMV2009. NT and JMV2009 were incubated at various time points in rat plasma. After protein precipitation, the supernatant was analyzed on HPLC/UV-MS as a ratio of the area under the curve (AUC) of the intact peptide over the area under the curve (AUC) of an internal standard.

Finally, we assessed the plasma stability of this novel neurotensin-like compound bearing a proline substitute. We incubated JMV2009 for various time points in rat plasma, and after protein precipitation and centrifugation, the intact remaining peptide was dosed by HPLC/UV-MS. We observed that JMV2009 possesses a plasma half-life of 6.24 ± 2.9 min, compared to 1.49 ± 0.4 min for the native NT (Fig. 4).

### 1.4 Signalling signature of JMV2009 at NT receptors

**Fig. 5:**
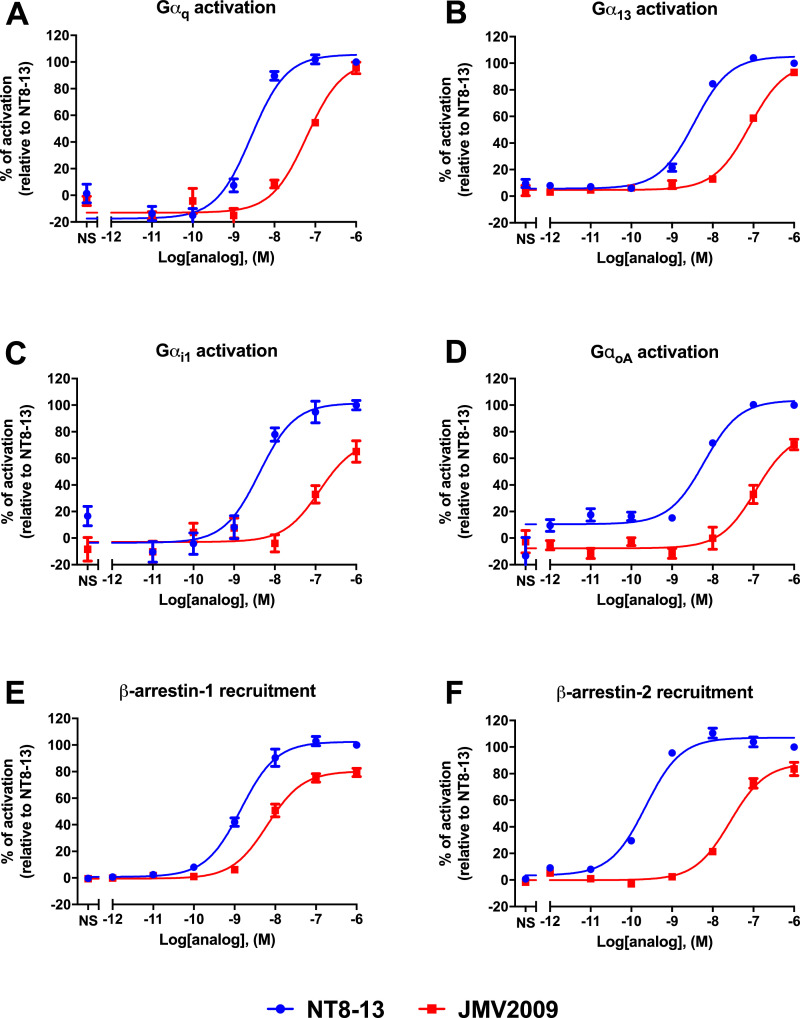
Effect of NT(8–13) and JMV2009 on NTS1 signalling. Activation of Gα_q_ (A), Gα_13_ (B), Gα_i1_ (C), and Gα_oA_ (D) after stimulation of CHO-K1 cells expressing hNTS1 and the G protein BRET-based biosensors. Recruitment of β-arr1 (E), and β-arr2 (F) upon activation of CHO-K1 cells transfected with hNTS1-GFP10 and β-arr1/2-Rluc biosensors.

We next assessed the signalling signature of this novel neurotensinergic compound, JMV2009, in comparison with the hexapeptide C-terminal fragment of neurotensin, NT(8–13). We used a bioluminescence resonance energy transfer-based assay to monitor the effect of NT(8–13) and JMV2009 on the activation of four G proteins known to be activated by NTS1 (Gα_q_, Gα_13_, Gα_i1_, and Gα_oA_) as well as the two β-arrestins (β-arr), also known to be recruited by NTS1 upon activation [5]. We observed a concentration-dependent response of NT(8–13) and JMV2009 for all G protein and β-arr pathways monitored (Fig. 5). A non-linear regression fit in Graphpad Prism has been used to determine the potency values (EC_50_) of NT(8–13) and JMV2009 (Table 2).

**Table 2:** Potency values of NT and JMV2009 at the NTS1 receptor.

	EC_50_ Gα_q_, nM	EC_50_ Gα_13_, nM	EC_50_ Gα_i1_, nM	EC_50_ Gα_oA_, nM	EC_50_ βarr1, nM	EC_50_ βarr2, nM
**NT8–13**	2.7 ± 0.6	3.6 ± 0.4	4.2 ± 1.4	6.2 ± 1.1	1.4 ± 0.2	0.22 ± 0.03
**JMV2009**	61.9 ± 15	80.4 ± 10	125 ± 72	114 ± 38	6.4 ± 9	27 ± 4.3

Values are expressed as EC_50_ ± SEM of at least three independent determinations.

**Fig. 6:**
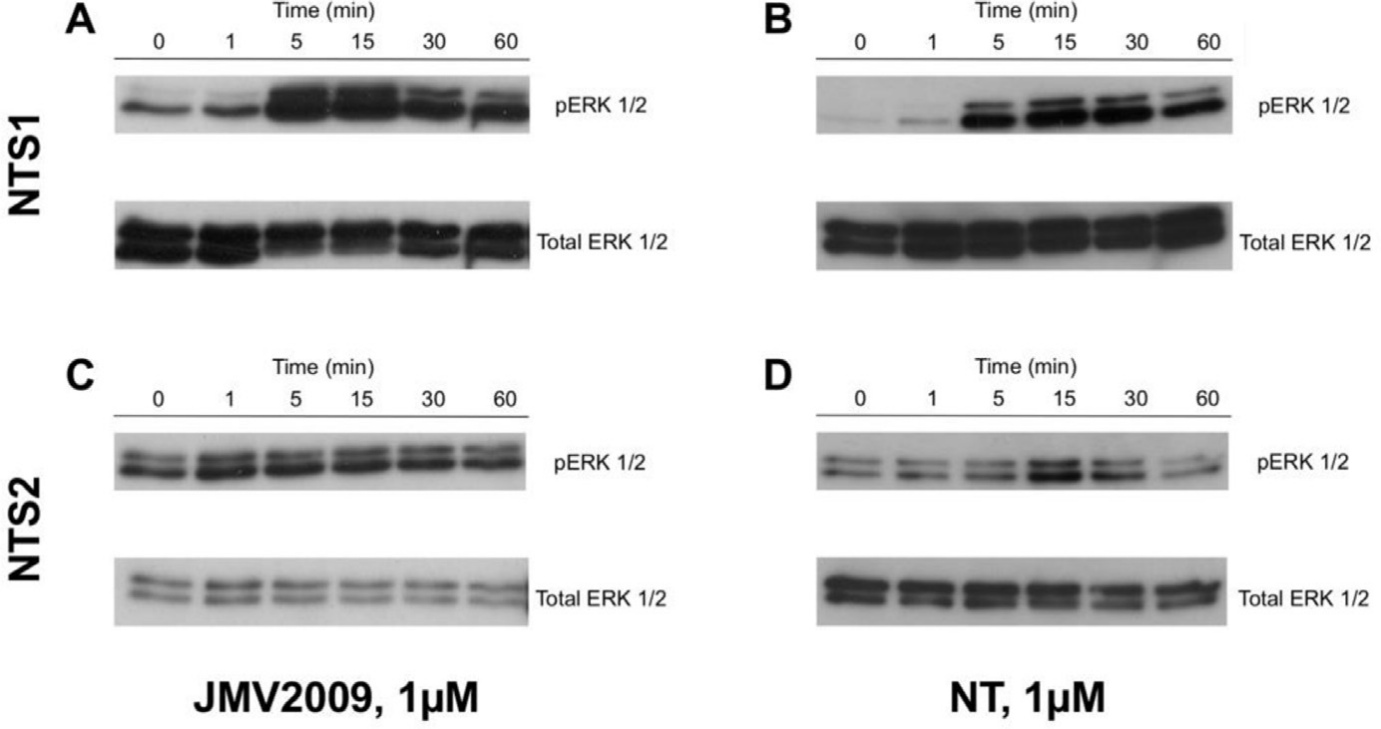
Phosphorylation of p42/p44 after stimulation with NT or JMV2009. Cells stably expressing either NTS1 or NTS2 were serum-starved for 24 h before stimulation with either 1 µM NT or JMV2009. Western blots represent immunoreactivity against phosphorylated p42/p44 (pERK1/2) or total p42/p44 (Total ERK1/2) proteins. Data represent CHO-K1 cells stably expressing NTS1 stimulated with JMV2009 (A) or NT (B) or 1321N1 cells stably expressing NTS2 stimulated with JMV2009 (C) or NT (D).

We further evaluated the ability of JMV2009 to induce an activation of the mitogen-activated protein kinases (MAPK) pathway after incubation at various time points with cells stably expressing either the NTS1 or NTS2 receptor. Thus, we performed western blots to monitor the phosphorylation of p42/p44 proteins (ERK 1/2) after stimulation with 1 µM of NT or JMV2009, as previously described by Gendron, et al. [6] We report here the time-dependent phosphorylation of p42/p44 proteins by the JMV2009 (Fig. 6).

**Fig. 7:**
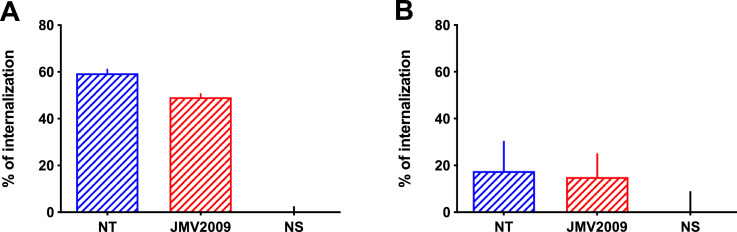
Internalization of NT receptors following stimulation with NT or JMV2009. Internalization of HA-hNTS1 receptor (A) or HA-hNTS2 receptor (B) monitored by cell-surface ELISA following a 60-min incubation period of transfected CHO-K1 with 1 µM of NT or JMV2009.

We finally investigated the ability of JMV2009 to trigger the internalization of NT receptors using a cell-surface ELISA assay, after stimulation of CHO-K1 cells transfected with the HA-tagged human NTS1 or NTS2 receptors. We found that JMV2009 was able to promote the internalization of both NT receptors (Fig. 5 and Table 3) after a 1 h-incubation period (Fig. 7).

**Table 3:** Internalization of NT receptors following stimulation with NT or JMV2009.

	NTS_1_ internalization,%	NTS_2_ internalization,%
**NT**	59.4 ± 1.5	17.7 ± 12
**JMV2009**	49.1 ± 1.4	15.1 ± 9.8

Values are expressed as mean ± SEM of at least three independent determinations.

## 2 Experimental design, materials, and methods

### 2.1 Materials

Supplements and media for cell culture are from Wisent (St-Bruno, QC, Canada). Cells stably expressing NTS1 (CHO-K1, ES-690-C) and NTS2 (1321N1, ES-691-C) as well as radiolabeled neurotensin are from PerkinElmer (Billerica, MA). CHO-K1 cells are from the American Type Culture Collection (CCL-61 from ATCC, Manassas, VA). Chemicals are from Fisher Scientific (Ottawa, ON, Canada) unless stated otherwise. Neurotensin 1–13 and the hexapeptide neurotensin 8–13 are synthesized by the peptide synthesis core facility of the Institut de Pharmacologie de Sherbrooke (https://www.usherbrooke.ca/ips/fr/plateformes/).

### 2.2 JMV2009 synthesis

Leucine residue-preloaded Wang resin was purchased from Novabiochem; amino acids bearing Fmoc-protection were obtained from ISIS Biotech. HBTU, HOBt, DIEA, TEA and piperidine were purchased from Aldrich. Acetonitrile and trifluoroacetic acid (TFA) were from Merck. ESI-MS was performed on a Micromass Platform II quadrupole mass spectrometer (Micromass) coupled with an HPLC.

Reverse phase analytical chromatograms were obtained using a C18 column (3.5 μm, 4.6 × 50 mm), coupled to a UV–Vis detector with a linear gradient of acetonitrile in water from 0 to 100% in 15 min at a flow of 1 mL/min. Retention time (*t_R_*) are given in minutes.

Waters Delta-Prep 4000 chromatography equipped with a 214 nm UV detector and mounted with a Delta-Pak C18 column (40 × 100 mm, 15 µm, 100 Å) was used as a preparative set-up with a flow rate of 50 mL min^−1^ of a binary eluent system of A: H_2_O, TFA 0.1% / B: CH_3_CN, TFA 0.1%.

Automated solid-phase peptide synthesis with a PerkinElmer ABI433A automatic synthesizer was used for the NT hexapeptide on a 0.25 mmol scale starting with Wang resin loaded with a leucine residue (loading 0,84 mmol/g) as previously described [7]. HBTU/HOBt (0.45 M) was used as coupling reagent with a 4-time excess of Fmoc-protected amino acid (1 mmol). Fmoc-Sip-OH has been synthesized according to published procedures [2,3]. piperidine:DMF (20:80) was used for deprotection and deprotection steps were followed using conductimetry. DMF with DIEA (2 M) as base were used during the 30-min coupling steps. Resin was washed between coupling steps using DMF and DCM. TFA:anisole 8:2 mixture was used for the final deprotection and cleavage for 3 h. The resin was washed extensively with DCM and filtered over cotton wool. Residual TFA was removed using hexane co-evaporation under vacuum. The residue was subsequently precipitated as a TFA salt and the solid precipitate was dried under vacuum before purification on preparative HPLC on C18. These conditions afforded the expected peptide (JMV2009) in 74% yield (210 mg of TFA salt), after purification. *t*_R_ =17.2 min (20 – 50% B, 30 min, C18). ES-MS [M+H]^+^ 805,7. F: 146–148 °C.

### 2.3 Cell culture

Cell lines stably expressing the human NTS1 receptor were cultured in DMEM/F12. NTS2-expressing cells were cultured in DMEM. Culture media were supplemented with 10% FBS, 100 U/ml penicillin-streptomycin, 2 mM l-glutamine, 20 mM HEPES, and 0.4 mg/mL of G418. CHO-K1 cells were cultured in the same DMEM/F12 as NTS1 cells but without G418 supplementation. Cells were kept at 37 °C under 5% CO_2_. All cell lines were used below passage 25.

### 2.4 Radioligand binding experiments

Binding experiments were carried out on freshly prepared membrane homogenates as previously described [4]. Competition radioligand binding experiments were performed by incubating cell membranes with ^125^I-[T*yr*^3^]-NT (specific activity of 2200 Ci/mmol) and different concentrations of ligands (ranging from 10^−11^ to 10^−5^ M) for a hour at room temperature. All binding data were plotted and fitted by using the One site – Fit Log(IC_50_) of Prism v7.0a (GraphPad, La Jolla, CA, USA) and represent the mean ± SEM of three separate determinations.

### 2.5 Plasma stability

#### 2.5.1 Plasma preparation

*Animals*: Adult male Sprague-Dawley rats (200–225 g; Charles River Laboratories, St-Constant, Quebec, Canada) were given free access to food and water and maintained in a 12 h light / 12 h dark cycle.

*Blood sampling and preparation of plasma*: Plasma was sampled from anesthetized rats by cardiac puncture in 4.5 mL plasma separating tubes coated with lithium heparin (from BD). Tubes were then centrifuged at 2500 rpm for 15 min at 4°C to separate the plasma from the blood cells. Plasma was stored at −80°C in 500 µL aliquots until use.

#### 2.5.2 Plasma stability assay

Rat plasma (27 µL) and 1 mM aqueous solution of ligand (6 µL) were incubated at 37 °C for 5, 10, 15, and 20 min. The reaction was stopped by adding 70 µL of CH_3_CN. After vortexing and centrifugation at 15,000 g for 20 min at 4 °C, the supernatant was analyzed by HPLC/UV (λ=230 nm). 6 µL of 1 mM solution of Fmoc-Gly-was added in each sample as an internal standard for quantification. Ratio between AUC of test compound and AUC of Fmoc-Gly-was used to determine remaining test compound percentage. One-phase decay non-linear regression from Prism v7.0a was used to determine the half-life. Each point represent the mean ± SEM of three independent determinations.

### 2.6 BRET-based assays for the activation of G proteins and recruitment of β-arrestins

#### 2.6.1 G protein activation

BRET-based biosensors used in this article directly measure the dissociation of Gα and Gγ protein subunits, and were kindly provided by Dr. Michel Bouvier (Department of Biochemistry and IRIC, Université de Montréal, Montréal, QC, Canada), as a member of the CQDM-funded research team (Drs. M. Bouvier, T.E. Hébert, S.A. Laporte, G. Pineyro, J.-C. Tardif, E. Thorin and R. Leduc). The assays were performed as previously described [8]. Briefly, 1.5  ×  10^6^ CHO-K1 cells were seeded into 55 mm^2^ cell culture dishes and transfected 24 hours later. The cells were transfected with either of the following biosensor couples: hNTS1, Gα_q_-RlucII, or Gα_13_-RlucII, or Gα_i1_-RlucII, or Gα_oA_-RlucII, together with Gβ_1_ and Gγ_1_-GFP^10^ as described [5]. On the final day of the experiment, cells were washed with 100 μL of PBS and stimulated with increasing concentrations of NT(8–13) or JMV2009 prepared in HBSS containing 20 mM HEPES. The cells were then stimulated with 5 µM of coelenterazine 400A (GoldBio, St-Louis, MO, USA), incubated at 37  °C for 5 minutes, and read on a GENios Pro plate reader (Tecan, Durham, NC, USA) using a BRET^2^ filter set (410 nm and 515 nm emission filters).

For each well, a BRET2 ratio was determined by dividing the GFP10-associated light emission by RlucII-associated light emission. The data was subsequently normalized relative to NT(8–13); values for non-treated cells were set as 0% pathway activation, and those for cells treated with 1 μM NT(8–13) were set as 100% pathway activation.

#### 2.6.2 β-arrestin recruitment

The monitoring of β-arrestin recruitment was done by the transient transfection of CHO-K1 cells with plasmids containing cDNAs encoding hNTS1-GFP^10^ and RlucII-β-arrestin 1 or 2. The same protocol as the one used for G protein activation was then used except that incubation time before luminescence reading was increased to 15 min.

### 2.7 Western blot analyses of ERK1/2 activity

Cells stably expressing NTS1 or NTS2 were grown for 48 h in complete culture media before being incubated for 16 h in serum-free media. Cells were then stimulated with either NT or JMV2009. Aspiration of media and addition of ice-cold PBS blocked any further protein phosphorylation. Cells were then lysed in RIPA containing proteases and phosphatases inhibitors before being centrifuged at 8000 g for 15 min.

Separation, transfer and blotting steps were performed as described previously [6] using anti-phosphorylated ERK1/2 (Cell Signaling, cat# 4376S, lot 18, Danvers, MA; 1:1000, in TBS-T, 1% BSA) or anti-ERK1/2 (Cell Signaling, cat# 4695S, lot 28; 1:1000, in TBS-T, 1% BSA) as primary antibodies and HRP-conjugated anti-rabbit IgG from goat (Cell Signaling, cat# 7074S, lot 28; 1:5000, in TBS-T, 1% BSA) as secondary antibody for detection.

### 2.8 JMV2009-induced NT receptors internalization

Receptor internalization using CHO-K1 cells transiently transfected with either HA-NTS1 or HA-NTS2 was carried out as previously described in details [9], [10], [11]. Before ELISA detection, cells were washed with PBS and stimulated using 1 µM of NT or JMV2009 for 60 min at 37 °C in serum-free media. Cells were then washed with PBS and remaining ELISA steps form the detailed protocol were followed and unchanged. Absorbance was read at 450 nm and data were normalized according to the protocol.

## CRediT authorship contribution statement

**Élie Besserer-Offroy:** Conceptualization, Methodology, Validation, Formal analysis, Investigation, Writing - original draft, Writing - review & editing, Visualization. **Pascal Tétreault:** Conceptualization, Methodology, Validation, Formal analysis, Investigation, Writing - review & editing, Visualization. **Rebecca L Brouillette:** Investigation. **Adeline René:** . **Alexandre Murza:** Investigation. **Roberto Fanelli:** . **Karyn Kirby:** Investigation. **Alexandre Parent:** Investigation. **Isabelle Dubuc:** Investigation. **Nicolas Beaudet:** Conceptualization, Validation, Formal analysis. **Jérôme Côté:** Investigation. **Jean-Michel Longpré:** Conceptualization, Validation, Formal analysis. **Jean Martinez:** Supervision. **Florine Cavelier:** Writing - review & editing, Supervision, Funding acquisition. **Philippe Sarret:** Conceptualization, Validation, Formal analysis, Supervision, Funding acquisition.

## Declaration of Competing Interest

The authors declare that they have no known competing financial interests or personal relationships which have, or could be perceived to have, influenced the work reported in this article.

## References

[1] Tétreault P. (2020). Pain relief devoid of opioid side effects following central action of a silylated neurotensin analog. Eur. J. Pharmacol..

[2] Martin C. (2011). Resolution of protected silaproline for a gram scale preparation. Amino Acids.

[3] Vivet B., Cavelier F., Martinez J. (2000). Synthesis of silaproline, a new proline surrogate. Eur. J. Org. Chem..

[4] Hapău D. (2016). Stereoselective Synthesis of β-(5-Arylthiazolyl) α-Amino Acids and Use in Neurotensin Analogues. Eur. J. Org. Chem..

[5] Besserer-Offroy E. (2017). The signaling signature of the neurotensin type 1 receptor with endogenous ligands. Eur. J. Pharmacol..

[6] Gendron L. (2004). Low-affinity neurotensin receptor (NTS2) signaling: internalization-dependent activation of extracellular signal-regulated kinases 1/2. Mol. Pharmacol..

[7] Fanelli R. (2015). Synthesis and characterization in vitro and in vivo of (l)-(Trimethylsilyl)alanine containing neurotensin analogues. J. Med. Chem..

[8] Brouillette R.L. (2020). Cell-penetrating pepducins targeting the neurotensin receptor type 1 relieve pain. Pharmacol. Res..

[9] Besserer-Offroy, É., et al., Monitoring cell-surface expression of GPCR by ELISA. protocols.io, 2020.

[10] Gusach A. (2019). Structural basis of ligand selectivity and disease mutations in cysteinyl leukotriene receptors. Nat. Commun..

[11] Luginina A. (2019). Structure-based mechanism of cysteinyl leukotriene receptor inhibition by antiasthmatic drugs. Sci. Adv..

[12] Kilkenny C. (2010). Animal research: reporting *in vivo* experiments: the ARRIVE guidelines. Br. J. Pharmacol..

